# Quiescent and Proliferative Fibroblasts Exhibit Differential p300 HAT Activation through Control of 5-Methoxytryptophan Production

**DOI:** 10.1371/journal.pone.0088507

**Published:** 2014-02-11

**Authors:** Huei-Hsuan Cheng, Kai-Hsuan Wang, Ling-yun Chu, Tzu-Ching Chang, Cheng-Chin Kuo, Kenneth K. Wu

**Affiliations:** 1 Metabolomic Medicine Research Center, China Medical University, Taichung, Taiwan; 2 Graduate Institute of Clinical Medicine Science, China Medical University, Taichung, Taiwan; 3 Institute of Cellular and System Medicine, National Health Research Institutes, Zhunan, Miaoli, Taiwan; 4 Institute of Biotechnology, National Tsing Hua University, Hsin-Chu, Taiwan; Philipps University, Germany

## Abstract

Quiescent fibroblasts possess unique genetic program and exhibit high metabolic activity distinct from proliferative fibroblasts. In response to inflammatory stimulation, quiescent fibroblasts are more active in expressing cyclooxygenase-2 and other proinflammatory genes than proliferative fibroblasts. The underlying transcriptional mechanism is unclear. Here we show that phorbol 12-myristate 13-acetate (PMA) and cytokines increased p300 histone acetyltransferase activity to a higher magnitude (> 2 fold) in quiescent fibroblasts than in proliferative fibroblasts. Binding of p300 to cyclooxygenase-2 promoter was reduced in proliferative fibroblasts. By ultrahigh-performance liquid chromatography coupled with a quadrupole time of flight mass spectrometer and enzyme-immunoassay, we found that production of 5-methoxytryptophan was 2–3 folds higher in proliferative fibroblasts than that in quiescent fibroblasts. Addition of 5-methoxytryptophan and its metabolic precursor, 5-hydroxytryptophan, to quiescent fibroblasts suppressed PMA-induced p300 histone acetyltransferase activity and cyclooxygenase-2 expression to the level of proliferative fibroblasts. Silencing of tryptophan hydroxylase-1 or hydroxyindole O-methyltransferase in proliferative fibroblasts with siRNA resulted in elevation of PMA-induced p300 histone acetyltransferase activity to the level of that in quiescent fibroblasts, which was rescued by addition of 5-hydroxytryptophan or 5-methoxytryptophan. Our findings indicate that robust inflammatory gene expression in quiescent fibroblasts vs. proliferative fibroblasts is attributed to uncontrolled p300 histone acetyltransferase activation due to deficiency of 5-methoxytryptophan production. 5-methoxytryptophan thus is a potential valuable lead compound for new anti-inflammatory drug development.

## Introduction

Recent studies indicate that fibroblasts exiting the cell cycle (quiescent cells) exhibit distinct metabolic activity and possess unique genetic expression profile different from those of proliferative fibroblasts [1], [2]. These cellular and metabolic features of quiescent fibroblasts imply that quiescent cells may have specific cellular functions. Our recent report reveals that quiescent fibroblasts are more active in mounting robust inflammatory responses than proliferative fibroblasts [3]. Quiescent fibroblasts express abundant COX-2 proteins in response to phorbol 12-myristate 13-acetate (PMA), interleukin 1β (IL-1β), tumor necrosis factor α (TNFα) and lipopolysaccharide (LPS) [4]. COX-2 expression is reduced by ∼50% in proliferative fibroblasts compared to that in quiescent fibroblasts. Our results further show that the differential COX-2 protein expression in quiescent vs. proliferative fibroblasts occur at the transcriptional level [4]. PMA- and proinflammatory mediator-induced COX-2 promoter activity in proliferative fibroblasts was ≦ 50% of that in quiescent fibroblasts [4]. As COX-2 is a major mediator of inflammation, the results suggest that quiescent fibroblasts are actively involved in inflammation. Once quiescent fibroblasts are driven into cell cycle and undergo proliferation, they develop a mechanism to control COX-2-mediated inflammatory responses.

Restricted proinflammatory mediator-induced COX-2 transcriptional activation in proliferative fibroblasts was thought to be contributed by soluble factors (named cytoguardin) released into conditioned medium [5]. Conditioned medium of quiescent fibroblasts did not exhibit COX-2 suppressing activity suggesting that quiescent fibroblasts do no release cytoguardin into the medium. By using comparative metabolomic analysis, we have recently resolved the chemical nature of cytoguardin, which belongs to a novel tryptophan metabolite, 5-methoxytryptophan (5-MTP) [6]. Proliferative fibroblasts produce 5-MTP via two enzymatic steps: (1) tryptophan hydroxylase-1 (TPH-1) converts L-tryptophan to 5-hydroxytryptophan (5-HTP) and (2) hydroxyindole O-methyltransferase (HIOMT) converts 5-HTP to 5-MTP [6]. 5-MTP is active in suppressing COX-2 in cancer cells. However, it is not clear whether 5-MTP suppresses COX-2 expression in quiescent fibroblasts nor is it known whether quiescent fibroblasts are defective in producing 5-MTP.

COX-2 transcriptional activation by PMA, proinflammatory cytokines and LPS depends on concurrent binding of several transactivators such as NF-κB, C/EBPβ, AP-1, and CREB/ATF2 to the core promoter region of COX-2 gene [7], [8]. Restricted COX-2 promoter activation in proliferative vs. quiescent fibroblasts stimulated with proinflammatory mediators could be due to selective inhibition of a transactivator or a global suppression of multiple transactivator binding to COX-2 promoter. In order to elucidate the underlying transcriptional mechanism, we analyzed transactivators binding to COX-2 promoter by chromatin immunoprecipitation (ChIP) and streptavidin-agarose pulldown (SAP) assays and found a global reduction of transactivators binding when fibroblasts enter into proliferation. Transcriptional coactivator, p300, contains a histone acetyltransferase (HAT) domain which at basal condition is inactive due to blockade by autoinhibitory elements [9], [10]. Once activated, p300 HAT acetylates histones to open the chromatin structure for transactivator binding and acetylates myriad transactivators including NF-κB, C/EBPβ, AP-1, and CREB-2 to enhance the transactivator binding to promoters [11], [12]. Since proinflammatory mediator-induced COX-2 transactivation was reported to require p300 coactivator [13], [14], these preliminary data led us to postulate that restriction of COX-2 response to inflammatory stimuli in proliferative vs. quiescent fibroblasts is due to differential control of p300 HAT by 5-MTP.

Our experimental results from this study reveal that proinflammatory mediators induce robust p300 histone acetyltransferase (HAT) activation in quiescent fibroblasts which declined in proliferative fibroblasts. Contrary to production and release of abundant 5-MTP into the extracellular milieu by proliferative fibroblasts, quiescent fibroblasts produced little 5-MTP which is correlated with uncontrolled p300 HAT activation and COX-2 expression. Addition of 5-MTP reduced the p300 HAT activation in quiescent fibroblasts to the level in proliferative fibroblasts. Taken together, these data suggest that quiescent and proliferative fibroblasts exhibit differential p300 HAT activation through control of 5-MTP production.

## Results

### Global reduction of binding of key transactivators to COX-2 promoter in pFb

**Figure 1:**
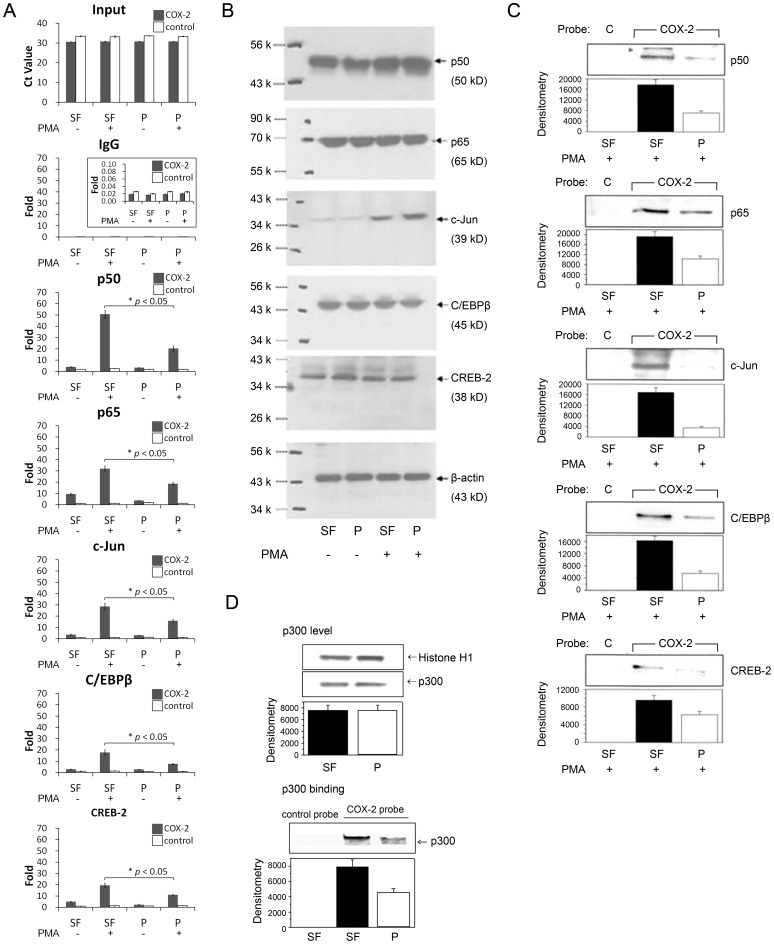
Binding of transactivators to COX-2 promoter is reduced in pFb vs. SF-Fb. SF-Fb and pFb were treated with or without PMA (100 nM) for 4 h. **A).** Binding of transactivators to COX-2 promoter was analyzed by ChIP. The precipitated promoter DNA was measured by qPCR. The results were expressed as ratio (fold) of COX-2 promoter precipitated by each transactivator antibody to input DNA. COX-2 promoter precipitated by an non-immune IgG was included as a negative control. “▪ COX-2” denotes core promoter region and “□ control” denotes negative region. The error bars denote mean ± SEM of three independent experiments (n = 3). **B).** Transactivator proteins in nuclear extracts were analyzed by Western blotting. **C)** and **D).** Analysis of concurrent binding of transactivators (C) and p300 (D) to a COX-2 probe (denoted “COX-2”) or control probe (denoted “C”) by streptavidin agarose pulldown assay. **D).** p300 proteins in nuclear extract were analyzed by Western blotting. The upper panel shows a representative blot and the lower panel, densitometry of the blot. The error bars denote mean ± SEM (n = 3). “SF” denotes SF-Fb and “P” denotes pFb.

To determine whether restricted COX-2 expression in proliferative fibroblasts (pFb) as compared to serum-free quiescent fibroblasts (SF-Fb) is due to reduced binding of a specific transactivator to COX-2 promoter, we analyzed binding of several functionally essential transactivators, i.e. NF-κB (p50/P65), C/EBPβ, c-Jun, CREB-2 to a core COX-2 promoter region by ChIP assay in pFb vs. SF-Fb treated with PMA. The precipitated promoter DNA was amplified and quantified with real-time qPCR. A COX-2 promoter region (−2150 ∼ −2030) without binding motifs for any of the transactivators was selected as a negative control. The data show that binding of all the transactivators was significantly lower in pFb than that in SF-Fb (Fig. 1A) while the nuclear transactivator protein levels were not different (Fig. 1B). Analysis of concurrent binding of transactivators by streptavidin-agarose pulldown assay confirmed global reduction of transactivators binding to a 500-bp COX-2 promoter probe in pFb vs. SF-Fb (Fig. 1C). These results indicate that restrained COX-2 transactivation in pFb is not due to blocking of a specific transactivator but attributed to suppression of a master regulator. We suspected that p300 is the target and, therefore, we next determined p300 binding to COX-2 promoter probe in pFb vs. SF-Fb. p300 binding to DNA-bound transactivators on COX-2 promoter was reduced in pFb compared to that in SF-Fb while nuclear p300 protein levels were not different (Fig 1D). These results suggest that p300 co-activator is involved in regulating the differential COX-2 trans-activation in pFb vs. SF-Fb.

### Deletion of p300 HAT domain impairs amplification of COX-2 promoter activation

**Figure 2:**
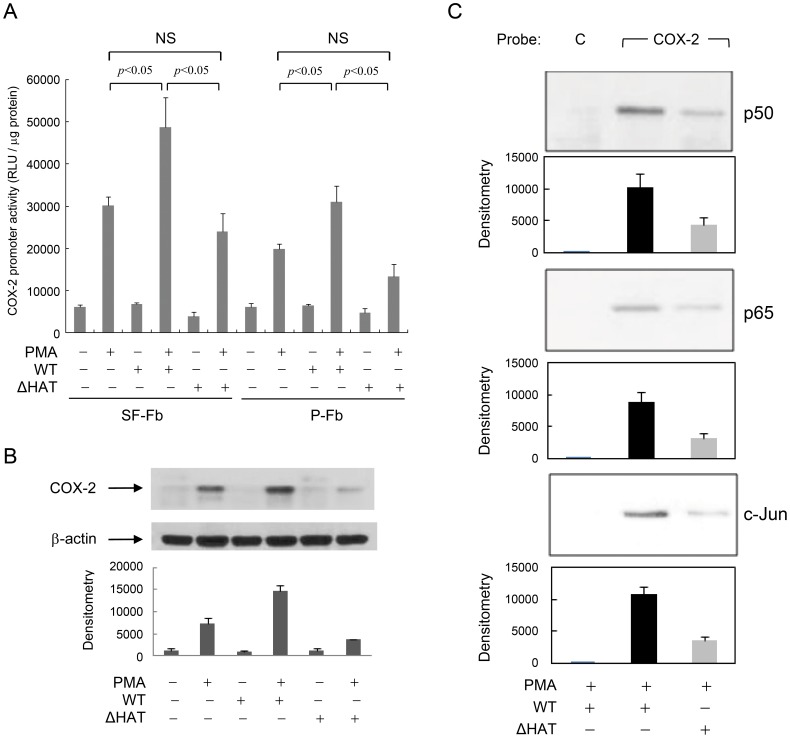
p300 HAT deletion mutant abrogates augmentation of PMA-induced COX-2 transcriptional activation. **A).** SF-Fb or pFb were transfected with full-length wild-type (WT) p300 or a p300 HAT domain deletion mutant (ΔHAT) followed by PMA-treatment. COX-2 promoter activity was analyzed. WT augmented PMA-induced COX-2 promoter activity while ΔHAT did not. “NS” denotes statistically non-significant. **B).** SF-Fb were transfected with WT or ΔHAT followed by PMA treatment. COX-2 proteins were analyzed by Western blotting. The upper panel shows a representative Western blot and the lower panel the densitometry. Error bars refer to mean ± SEM (n = 3). **C).** SF-Fb were transfected with WT or ΔHAT followed by PMA treatment. Nuclear extracts were prepared and binding of the nuclear transactivators to a core COX-2 promoter probe was analyzed by SAP assay. The error bars refer to mean ± SEM of three independent experiments. “C” denotes control probe and “COX-2”, COX-2 promoter probe.

We previously reported that inhibition of p300 histone acetyltransferase (HAT) with adenoviral E1A abrogates COX-2 transcriptional activation in SF-Fb stimulated with PMA and proinflammatory cytokines [14]. Furthermore, transcription of wild-type (WT) p300 amplified COX-2 transcriptional activation, which is severely attenuated when SF-Fb were transfected with a HAT domain deletion mutant (ΔHAT) [14]. To determine whether p300 HAT is involved in COX-2 transcription in pFb and SF-Fb, we transfected SF-Fb and pFb with WT p300 or ΔHAT and analyzed PMA-induced COX-2 promoter activity. Consistent with our previous report [14], WT p300 transfection augmented PMA-induced COX-2 promoter activity whereas the HAT domain deletion mutant, ΔHAT, did not (Fig. 2A). WT p300 transfection also augmented COX-2 promoter activity in pFb albeit to a lesser extent and ΔHAT transfection slightly reduced PMA-induced COX-2 promoter activity compared to untransfected pFb (Fig. 2A). Deletion of the HAT domain also failed to increase COX-2 protein expression in SF-Fb (Fig. 2B). To ensure that p300 HAT is required for PMA-induced transactivator binding to COX-2 promoter, we transfected SF-Fb with WT p300 or ΔHAT and evaluated the concurrent binding of transactivators to the COX-2 promoter probe by streptavidin-agarose pulldown assay. Compared to that in WT-transfected cells, binding of all the transactivators was diminished in PMA-treated ΔHAT-transfected SF-Fb (Fig. 2C). These results indicate that p300 HAT plays an essential role in proinflammatory mediator-induced COX-2 transcription in pFb and SF-Fb.

### Proinflammatory mediator-induced p300 HAT activities in SF-Fb and pFb

**Figure 3:**
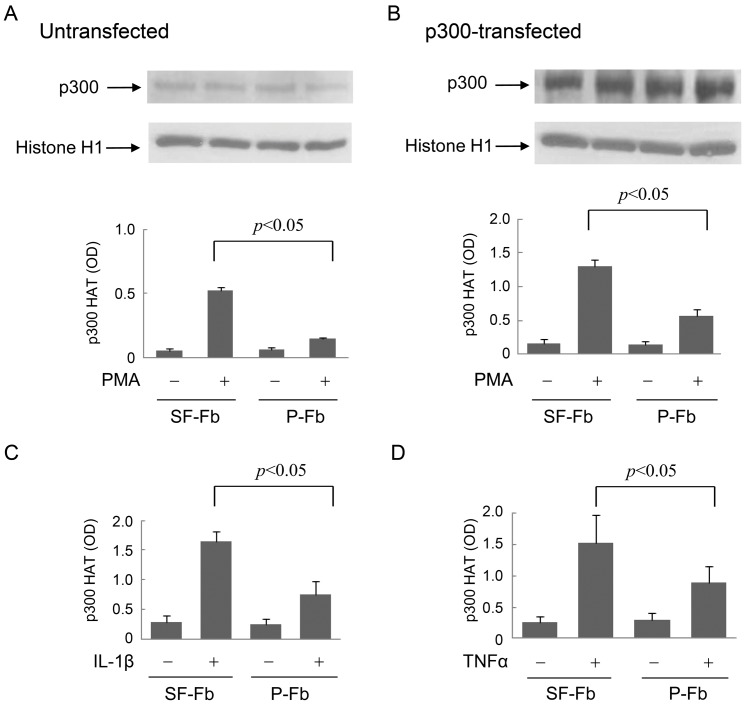
p300 HAT activation is decreased in pFb vs. SF-Fb. **A).** PMA-induced p300 HAT activity in untransfected native cells. Upper panel shows p300 proteins analyzed by Western blotting and lower panel, p300 HAT activity. **B).** p300 HAT activity in p300-transfected fibroblasts. Upper panel shows p300 proteins in Fb transfected with full-length p300 vectors and lower panel PMA-induced p300 HAT activity in the p300 overexpressing cells. Nuclear histone H1 was used as a loading control. **C)** and **D).** IL-1β- and TNFα-induced p300 HAT activities in p300-transfected SF-Fb or pFb. Each error bar denotes mean ± SEM (n = 3).

To provide direct evidence for a more robust activation of p300 HAT in quiescent fibroblasts than in proliferative fibroblasts, we isolated p300 by immunoprecipitation from SF-Fb and pFb treated with and without PMA and analyzed the HAT activity. As anticipated from the autoinhibition of p300 HAT, the basal p300 HAT activity was very low in SF-Fb as well as in pFb (Fig. 3A). PMA increased p300 HAT activity to a greater extent in SF-Fb than in pFb (Fig. 3A). PMA-stimulated p300 HAT activity in SF-Fb was augmented by p300 transfection (Fig. 3B). Although PMA-induced p300 HAT activity in pFb was also augmented by p300 overexpression, the extent of increase was less than that in SF-Fb (Fig. 3B). IL-1β or TNF-α stimulated p300 HAT activity differentially in pFb vs. SF-Fb and the extent of stimulation and magnitude of difference between SF-Fb and pFb were comparable to PMA (Fig. 3C & 3D).

**Figure 4:**
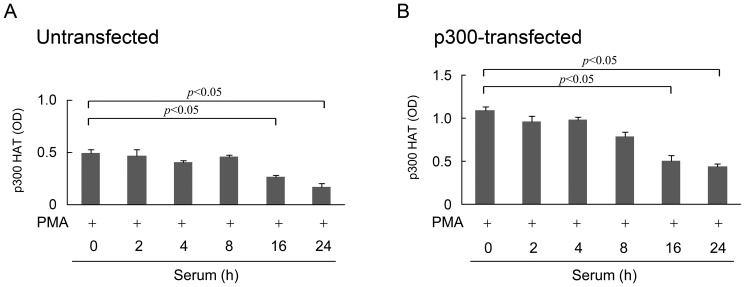
Time dependent reduction of PMA-induced p300 HAT activity in quiescent fibroblasts replenished with serum. **A).** Untransfected fibroblasts and **B).** p300-transfected fibroblasts. SF-Fb (denotes 0 h) were washed and incubated in fresh medium containing 2.5% FBS. At the indicated time points, cells were treated with PMA (100 nM) for 4 h and p300 HAT activity was measured. Each error bar denotes mean ± SEM (n = 3).

To determine whether reduction of p300 HAT activation in proliferative fibroblasts is correlated with cell cycle progression, we measured PMA-induced p300 HAT activity in the 24 h SF-Fb washed and replenished with 2.5% FBS for various time points. PMA-induced p300 HAT activity declined at 16 h after serum readdition in untransfected (Fig. 4A) as well as in p300-transfected cells (Fig. 4B) which is correlated with entrance into the S-phase of cell cycle and decline of COX-2 expression as previously described [4]. These results suggest that cell cycle progression and cell proliferation engendered restriction of p300 HAT activation induced by PMA and proinflammatory cytokines.

### Suppression of p300 HAT activation in SF-Fb by soluble factors released from pFb

**Figure 5:**
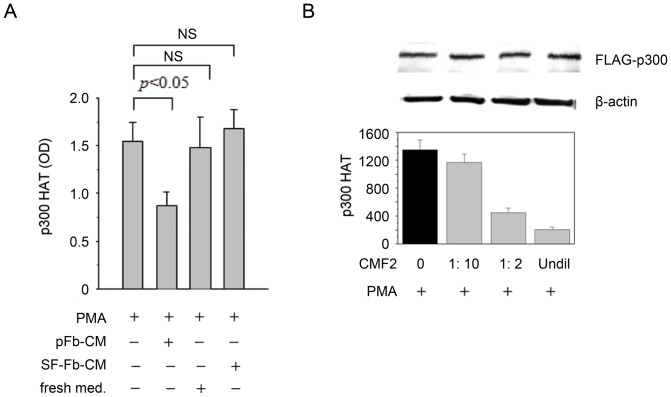
p300 HAT activity in SF-Fb is suppressed by conditioned medium (CM) and CMF2. **A).** SF-Fb were washed and incubated with CM from proliferative fibroblasts (pFb-CM), from quiescent fibroblasts (SF-Fb-CM) or fresh medium containing 2.5% FBS (fresh medium) for 24 h followed by treatment with PMA (100 nM) for 4 h. Each error bar denotes mean ± SEM (n = 3). **B).** Diluted & undiluted CMF2 prepared from pFb-CM was incubated for 24 h with washed SF-Fb transfected with p300 vectors. PMA-induced p300 HAT activity was measured. “Undil” denotes undiluted CMF2 while “1∶10” and “1∶2” denote dilution of CMF2 with medium. “0” denotes without CMF2. Each error bar denotes mean ± SEM (n = 3).

Conditioned medium of pFb (pFb-CM) was reported to contain soluble factors (named cytoguardin) which suppress COX-2 transcriptional activation by proinflammatory mediators [5]. We determined whether pFb-CM exerted an effect on p300 HAT activation. pFb-CM inhibited PMA-induced p300 HAT activation in SF-Fb by ∼50% while SF-CM did not (Fig. 5A). Nor did the control medium (Fig 5A). We prepared CMF2 fraction from pFb-CM by several purification steps and tested its ability to control p300 HAT activation in SF-Fb. Lyophilized CMF2 was serially diluted and added to washed SF-Fb. CMF2 inhibited PMA-induced p300 HAT in a concentration dependent manner (Fig. 5B) These results suggest that cytoguardins released by pFb possess inhibitory action on p300 HAT activation.

**Figure 6:**
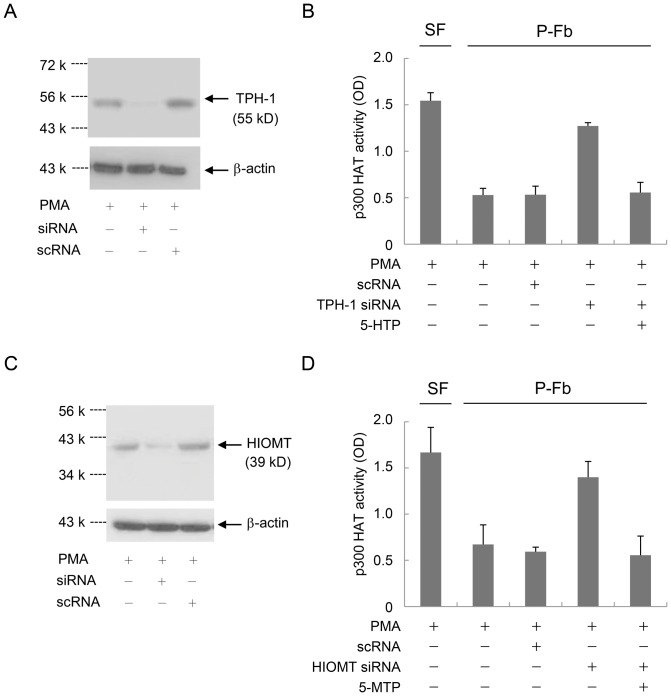
p300 HAT suppression in pFb is reversed by silencing of 5-MTP synthetic enzymes. 5-MTP synthesis in pFb (P-Fb) is catalyzed by tryptophan hydroxylase-1 (TPH-1) and hydroxyindole o-methyltransferase (HIOMT). **A)** and **B).** pFb transfected with TPH-1 siRNA or control scRNA were treated with PMA for 4 h. **A).** TPH-1 proteins were analyzed by Western blotting. **B).** p300 proteins were isolated by IP and HAT activity was measured. siRNA but not scRNA treatment resulted in p300 HAT elevation to a level comparable to that in SF-Fb. Addition of 5-HTP (10 µM) for 30 min restored p300 HAT suppression. Error bars are mean ± SEM (n = 3). **C)** and **D).** pFb transfected with HIOMT siRNA or scRNA were treated with PMA. **C).** HIOMT protein analysis by Western blotting. **D).** p300 HAT analysis. HIOMT siRNA abrogated p300 HAT suppression which was restored by addition of 5-MTP (10 µM) for 30 min. The error bars denote mean ± SEM (n = 3).

We have recently identified 5-MTP as a COX-2 suppressing cytoguardin [6]. 5-MTP production in pFb was shown to be catalyzed by tryptophan hydroxylase-1 (TPH-1) which converts L-tryptophan to 5-hydroxytryptophan (5-HTP) and hydroxyindole O-methyltransferase (HIOMT) which converts 5-HTP to 5-MTP [6]. To determine that 5-MTP production is responsible for control of p300 HAT activation, we silenced TPH-1 with siRNA (Fig. 6A) which abrogated p300 HAT suppression in pFb (Fig. 6B). PMA-induced p300 HAT activity in TPH-1 siRNA-treated pFb was increased to the level detected in SF-Fb (Fig. 6B). The control scRNA did not alter the p300 HAT in pFb (Fig. 6B). Interestingly, addition of 5-HTP restored p300 HAT suppression despite TPH-1 siRNA treatment (Fig 6B). HIOMT siRNA which inhibited HIOMT protein expression (Fig. 6C) also abrogated p300 HAT suppression in pFb and 5-MTP restored p300 HAT inhibition (Fig. 6D). These results confirm the essential role of 5-MTP production in conferring control of proinflammatory mediator-induced p300 HAT activation and COX-2 expression in proliferating fibroblasts.

### Quiescent fibroblasts are deficient in 5-MTP production

**Figure 7:**
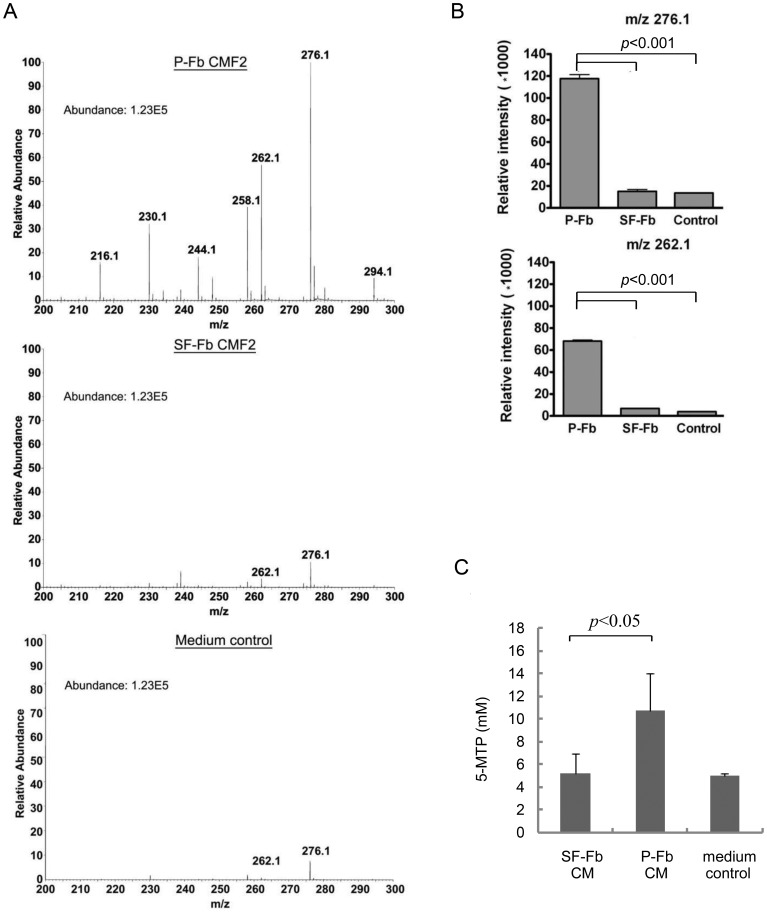
Deficient 5-MTP production in SF-Fb. **A).** Metabolomic analysis of CMF2 fraction of SF-Fb vs. pFb. CMF2 of the control medium was included as negative control. A representative mass spectra is shown. **B).** Intensity of m/z 272.1 and 262.1 from multiple experiments (n = 5). The error bars denote mean ± SEM. **C).** Measurement of 5-MTP in CM of SF-Fb vs. pFb by EIA. Medium control refers to fresh medium containing 2.5% fetal bovine serum.

As CM of SF-Fb fails to suppress proinflammatory mediator-induced COX-2 and p300 HAT activation, we suspected that quiescent fibroblasts could be deficient in 5-MTP production. To discern this, we compared the metabolomic profile of SF-Fb vs. pFb CMF2 using a high resolution UPLC-QTof mass spectrometry system. Consistent with our recent report, the mass spectra of pFb CMF2 revealed prominent m/z 276.1, m/z 262.1 and several other peaks at m/z 200-300 (Fig. 7A). The intensity of m/z 276.1 & 262.1 was greatly diminished in SF-Fb (Fig 7A) which was not different from that of medium control (Fig. 7A). The intensity of m/z 276.1 and m/z 262.1 in CMF2 of pFb were several-fold higher than that in CMF2 of SF-Fb or medium control (Fig. 7B). M/z 276.1 and m/z 262.1 were identified as 5-MTP and 5-HTP, respectively [6]. The metabolomic profiling suggests that SF-Fbs are deficient in 5-MTP production. Quantification of 5-MTP by EIA confirms a significantly lower 5-MTP level in SF-Fb CM than in pFb CM (Fig. 7C). 5-MTP in SF-Fb CM was not different from that in serum-containing medium control (Fig. 7C). These results indicate that quiescent fibroblasts produce very low level of 5-MTP.

### Suppression of SF-Fb p300 HAT activation by 5-HTP

**Figure 8:**
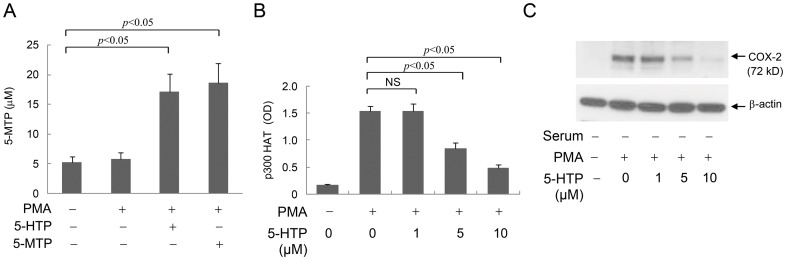
5-HTP inhibits p300 HAT activation and COX-2 expression. **A).** SF-Fb were treated with 5-HTP (10 µM) or 5-MTP (10 µM) for 30 min followed by PMA (100 nM) for 4 h. 5-MTP in the conditioned medium was measured by EIA. The error bars indicate mean ± SEM (n = 3). **B) and C).** SF-Fb were treated with 5-HTP at increasing concentrations (1–10 µM) for 30 min followed by PMA for 4 h. B). p300 HAT activity and C). COX-2 protein expression were analyzed. The error bars in B) refer to mean ± SEM (n = 3). The western blot shown in C) is representative of two experiments with similar results. 5-HTP inhibited p300 HAT activity in a concentration-dependent manner which is correlated with concentration-dependent COX-2 suppression.

As 5-HTP is an intermediate metabolite of 5-MTP synthesis, we determined whether 5-HTP exerts an effect on p300 HAT activation. Addition of 5-HTP to SF-Fb resulted in generation of 5-MTP (Fig. 8A). Quantitative analysis revealed that the added 5-HTP (10 µM) was completely converted to 5-MTP as addition of 10 µM 5-MTP yielded a comparable amount as 10 µM 5-HTP (Fig. 8A). We next evaluated the effect of 5-HTP on PMA-induced p300 HAT activity. 5-HTP inhibited PMA-induced p300 HAT activity in a concentration-dependent manner (Fig. 8B). Furthermore, 5-HTP inhibited PMA-induced COX-2 proteins in a concentration-dependent fashion correlating with p300 HAT inhibition (Fig. 8C). These results suggest that SF-Fb are capable of converting 5-HTP to 5-MTP.

### 5-MTP inhibits PMA-induced p300 HAT activation

**Figure 9:**
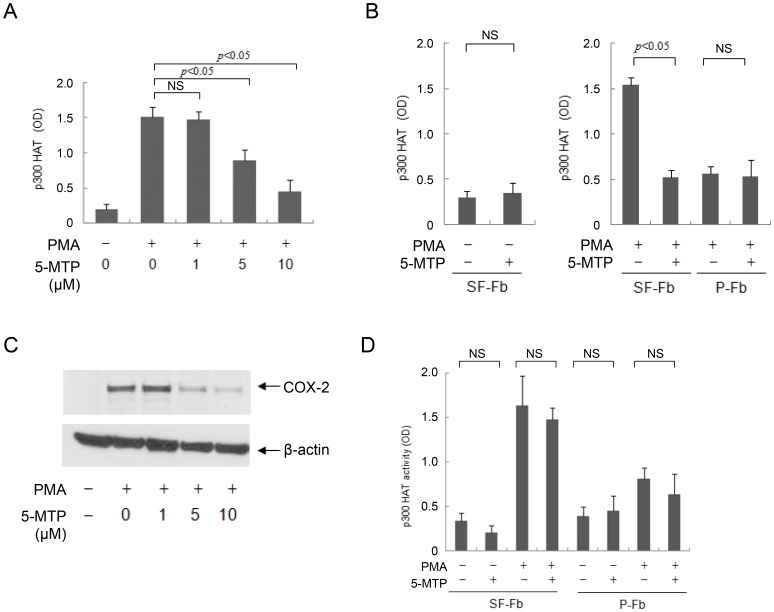
5-MTP blocks PMA-induced p300 HAT activity in SF-Fb. **A).** SF-Fb were pretreated with 5-MTP at indicated concentrations for 30 min followed by PMA for 4 h. p300 HAT activity was measured. The error bars show mean ± SEM (n = 3). **B).** SF-Fb were treated with 5-MTP (10 µM) in the absence of PMA (left panel) or presence of PMA (right panel). Right panel also shows treatment of pFb with or without 5-MTP followed by PMA. Each error bar refers to mean ± SEM (n = 3). “NS” denotes statistically non-significant. **C).** SF-Fb were pretreated with 5-MTP (10 µM) for 30 min followed by PMA (100 nM) for 4 h. COX-2 proteins were analyzed with Western blotting. This Western blot is representative of two experiments with similar results. **D).** SF-Fb or pFb were treated with or without PMA (100 nM) for 4 h. p300 proteins were isolated by IP, washed and treated with 5-MTP (10 µM) for 30 min. HAT activity was measured. The error bars refer to mean ± SEM (n = 3). “NS” denotes statistically non-significant.

To provide further evidence for the control of p300 HAT by 5-MTP, we evaluated the effect of 5-MTP on PMA-induced p300 HAT activity. 5-MTP inhibited PMA-induced p300 HAT activity in SF-Fb in a concentration-dependent manner (Fig. 9A) and at 10 µM it reduced p300 HAT activity by >50% without influencing the basal activity (Fig. 9B). By contrast, 5-MTP did not inhibit PMA-induced p300 HAT activity in pFb (Fig. 9B). Consistent with our previous report [6], 5-MTP inhibited PMA-induced COX-2 protein expression in a manner correlated with its inhibition of p300 HAT activation (Fig. 9C). It is worthy noting that 5-HTP inhibits p300 HAT activation and COX-2 expression at concentrations comparable to 5-MTP (Fig. 8B and C vs. Fig. 9A-C), consistent with the interpretation that 5-HTP exerts its effect via 5-MTP. Taken together, these findings confirm that robust p300 HAT activation and COX-2 expression in SF-Fb is attributed to deficiency of 5-MTP production whereas restrained p300 HAT activation and COX-2 expression in pFb is contributed by abundant 5-MTP production.

To determine whether 5-MTP has a direct effect on p300 HAT activation, we isolated p300 proteins by IP from SF-Fb and pFb treated with or without PMA, washed them and treated them with 5-MTP (10 µM). 5-MTP did not inhibit HAT activity of p300 proteins isolated from SF-Fb or pFb (Fig. 9D). It is, hence, unlikely that 5-MTP exerts a direct effect on p300 HAT activity.

### PKCδ is involved in signaling PMA-induced p300 HAT activation

**Figure 10:**
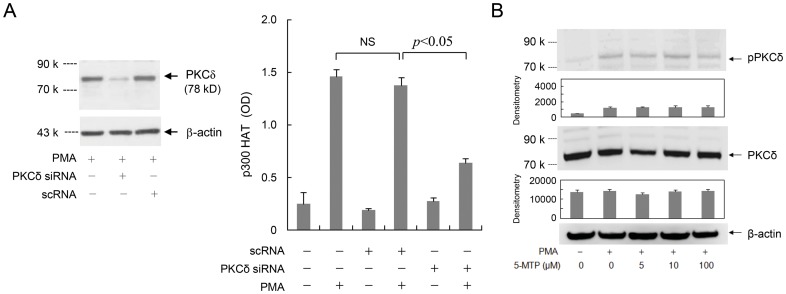
PKCδ signals PMA-induced p300 HAT activation. **A).** SF-Fb were transfected with a PKCδ siRNA or control scRNA followed by treatment with PMA (100 nM) for 4 h. p300 HAT was analyzed. Left panel: PKCδ proteins were analyzed by Western blotting. Right panel: Analysis of p300 HAT activity in siRNA vs. scRNA transfected SF-Fb. The error bars refer to mean ± SEM (n = 3). **B).** SF-Fb were treated with 5-MTP at increasing concentration followed by PMA (100 nM) for 4 h. Cell lysates were collected and the level of the indicated proteins was analyzed by Western blotting. “p-PKCδ” denotes phosphorylated PKCδ. Upper panels show representative blots and lower panel, densitometry of three experiments. Error bars refer to mean ± SEM (n = 3). β-actin was included as loading control.

In view of the previously reported data indicating that COX-2 transcriptional activation by PMA is mediated via PKCδ [3], we investigated the role of PKCδ in PMA-induced p300 HAT activation. We transfected SF-Fb with PKCδ siRNA or a control scRNA and analyzed PMA-induced p300 HAT activity. PKCδ siRNA abrogated the stimulatory effect of PMA while scRNA had no effect (Fig. 10A). We next determined whether PKCδ activation by PMA is suppressed by 5-MTP. 5-MTP did not inhibit PKCδ proteins (Fig. 10B), nor did it significantly alter phosphorylated PKCδ (pPKCδ) (Fig. 10B). These results support a crucial role of PKCδ in mediating PMA-induced p300 HAT activation. However, 5-MTP inhibits PMA-induced p300 activation by a mechanism independent of PKCδ.

## Discussion

The findings of this study provide evidence for a critical role of p300 HAT in governing the differential transcriptional activation of COX-2 by proinflammatory mediators in quiescent vs. proliferative fibroblasts. Robust expression of COX-2 in response to stimulation by PMA and cytokines in quiescent fibroblasts as compared to restrained COX-2 response in proliferative fibroblasts is correlated with differential p300 HAT activation in quiescent vs. proliferative fibroblasts. We have previously shown that p300 HAT is crucial for COX-2 transcriptional activation by proinflammatory mediators as inhibition of p300 HAT activation by adenoviral E1A abrogates the increase in COX-2 expression [14]. In this study, we compared the augmenting effect of transfection of WT vs a p300 HAT domain deletion mutant (ΔHAT) on PMA-induced COX-2 promoter activity, protein expression and transactivator binding to COX-2 promoter in SF-Fb vs. pFb. WT p300 transfection significantly augmented PMA-induced COX-2 promoter activity in SF-Fb as well as in pFb, although the magnitude of augmentation in pFb was lower than that in SF-Fb. The augmenting effect was abrogated in SF-Fb as well as in pFb when either cell was transfected with ΔHAT. Compared to untransfected SF-Fb or pFb, ΔHAT transfection slightly reduced the COX-2 promoter activity (Fig. 2A). PMA-induced COX-2 protein expression was also lower in ΔHAT transfected SF-Fb than in untransfected cells. Reduction in PMA-induced COX-2 expression in ΔHAT-transfected cells may be attributed to competition of wild-type p300 binding by the ΔHAT mutant. It may also be attributed to competition against other co-activators such as CREB-binding protein (CBP) binding. Taken together, these findings indicate a differential control of p300 HAT activation in quiescent vs. proliferative fibroblasts resulting in robust expression COX-2 in quiescent fibroblasts contrasted with a restrained COX-2 response in proliferative fibroblasts.

Little is known about the regulation of p300 HAT activation induced by proinflammatory mediators. Even less clear is the cell cycle-dependent control of p300 HAT activation. Our data shed lights on the control of p300 HAT activation by soluble factors released by proliferating fibroblasts. We have identified one of the factor as 5-MTP, a novel tryptophan metabolite recently reported by our laboratory [6]. We show by silencing the expression of 5-MTP synthetic enzymes, TPH-1 or HIOMT, that 5-MTP is endogenously produced to control p300 HAT activation in proliferating fibroblasts. By contrast, quiescent fibroblasts are relatively inactive in producing 5-MTP which accounts for robust p300 HAT activation in response to proinflammatory stimulation. Robust p300 HAT activation in quiescent fibroblasts is suppressed by exogenous addition of 5-MTP suggesting that in the inflammatory microenvironment where quiescent and proliferative fibroblasts co-exist, 5-MTP produced by proliferative fibroblasts plays a role in controlling quiescent fibroblast p300 HAT activation. It is interesting that exogenous 5-HTP suppresses p300 HAT activation to an extent comparable to 5-MTP. Since quiescent fibroblasts convert 5-HTP to 5-MTP, it may be assumed that quiescent fibroblasts possess active HIOMT and their defect in 5-MTP production may reside at the level of TPH-1. Further studies are needed to elucidate the underlying enzymatic defect responsible for poor 5-MTP production.

5-MTP suppresses PMA-induced COX-2 expression in SF-Fb in a concentration-dependent manner corresponding to its inhibition of PMA-induced p300 HAT activation. As p300 HAT is required to drive PMA-induced COX-2 expression, our results imply that 5-MTP inhibits the master regulator p300 HAT whereby it controls the expression of COX-2 expression. The mechanism by which 5-MTP inhibits proinflammatory mediator-induced p300 HAT activation is unclear. Our results indicate that 5-MTP does not exert a direct inhibitory action on p300 HAT. We show that PMA-induced p300 HAT activation is mediated via PKCδ. 5-MTP does not alter PKCδ protein levels nor does it significantly influence phosphorylated PKCδ, suggesting that 5-MTP inhibits PMA-induced p300 HAT activation by a mechanism independent of PKCδ interruption.

p300 is a transcriptional co-activator for the transcription of an array of genes exhibiting common promoter characteristics [15], [16]. More robust activation of p300 HAT in quiescent than in proliferative fibroblasts stimulated with PMA or cytokines is expected to result in more abundant expression of an array of genes besides COX-2 in quiescent than in proliferative fibroblasts. Microarray analysis of gene expression profiling in PMA-treated quiescent vs. proliferative fibroblasts revealed a higher level expression of more than 20 genes, mostly belonging to cytokine and mitogen family genes, in quiescent fibroblasts as compared with proliferative fibroblasts [3]. It is likely that the differential gene expression profile is due to differential control of p300 HAT in quiescent vs. proliferative fibroblasts by 5-MTP. Work is in progress to test this possibility.

Our findings suggest that quiescent fibroblasts actively participate in inflammatory responses through robust p300 HAT activation and p300-mediated proinflammatory gene expression. At resting state, most fibroblasts are quiescent and provide structural support for tissue integrity. The conventional view was that quiescent fibroblasts are not active responders to environmental changes. Recent studies have shown that tissue-bound quiescent fibroblasts are in fact dynamic and responsive to environmental cues [17], [18]. For example, upon skin or vascular injury, fibroblasts migrate from their residence to the injury sites where they undergo phenotypic changes and become proliferative and synthetic. The proliferative, synthetic fibroblasts are considered to be a major contributor of inflammation, fibrosis and tissue remodeling [18]. Our results suggest that proliferative fibroblasts play complex roles in inflammation. They contribute to inflammation by upregulating cytokine and extracellular matrix production. However, through 5-MTP production, they may cool down the inflammatory response by inhibiting the participating inflammatory cells such as macrophages and fibroblasts to produce proinflammatory cytokines. Thus, fibroblasts at quiescent and proliferative stages interact to regulate inflammatory responses. These *in vitro* results need to be confirmed by *in vivo* experiments.

Dysregulated p300 HAT activation was reported to be associated with human diseases such as cancer, cardiac hypertrophy, asthma and diabetes of which inflammation is a pivotal underlying pathophysiological process [19]–[22]. There are increasing interests to use p300 HAT as a target to develop new therapeutic agents. Several small-molecule inhibitors of p300 HAT were reported [23]–[25]. The reported chemicals inhibit basal p300 HAT activity. By contrast, 5-MTP does not inhibit basal p300 HAT activity but blocks proinflammatory mediator-induced p300 HAT activation. 5-MTP is better suited as a lead compound for developing new therapeutic agents to treat diverse inflammatory diseases as well as for prevention of inflammation-triggered cancer growth and metastasis.

## Materials and Methods

### Plasmids

A promoter region of human COX-2 gene (-891 to +9) was constructed into a luciferase reporter vector pGL3 as previously described [7]. Expression vectors containing wild-type (WT) full-length p300 (pCL.p300) (WT p300) and HAT-deleted mutant p300 (ΔHAT) were provided by Dr. Joan Boyes.

### Chemical reagents

5-MTP (#M4001), 5-HTP (#H9772) and PMA (#P8139) were purchased from Sigma-Aldrich, St. Louis, MO. IL-1β (#8900) and TNFα (#8902) were purchased from Cell Signaling Technology, Danvers, MA.

### Cell culture

Human foreskin fibroblasts (Fb) Hs68 were obtained from American Type Culture Collection (ATCC, Manassas, VA) and cultured in Dulbecco's modified Eagle's medium (DMEM) supplemented with 10% fetal bovine serum (FBS) and 1∶100 dilution of an antibiotic-antimycotic solution (Invitrogen, Grand Island, NY). Human fibroblasts are commonly used as a cell model to investigate genetic and biochemical changes accompanying all cycle progression [1]–[3]. We prepared proliferative and quiescent Fb as previously described [3], [4]. In brief, cultured Fb were washed and incubated in serum-free (SF) medium for 24 h. Analysis of cell cycle by flow cytometry shows that a vast majority (≧ 90%) of the 24 h SF fibroblasts are at G0/G1 [4]. Those cells are mostly quiescent and are designated SF-Fb. After washing, Fb were incubated in medium containing 2.5% FBS and at several time points up to 24 h, cells were treated with or without PMA (100 nM). We previously reported that Fb enter S-phase at 16 h after addition of FBS and by 24 h over 80% of cells are at S-phase or G2/M [4]. The 24 h serum-replenished Fb were designed proliferative fibroblasts (pFb).

### Analysis of COX-2 promoter activity

The promoter activity was determined by transient expression of the COX-2 promoter construct by a previously described method [7]-[9]. In brief, 4 µg of promoter vectors were mixed with 10 µl of lipofectamine 2000 (Invitrogen, Carlsbad, CA). The mixture was slowly added to cells in a 6-well plate and incubated for 24 h. After treatment, cells were lysed and luciferase activity was measured using an assay kit form Promega (Madison, WI) in a luminometer (TD 20/20).

### Streptavidin-agarose pulldown (SAP) assay

Binding of transactivators to COX-2 promoter was analyzed by SAP assay as previously described [26], [27]. A biotin-labeled double-stranded probe corresponding to COX-2 promoter sequence -30 to -508 was synthesized by Integrated DNA Technologies (Coralville, IA). A nonrelevant biotinylated sequence 5′- A G A G T G G T C A C T A C C C C C T C T G -3′ and a -30/-508 probe in which the κB site were mutated were included as a control [26]. The binding assay was performed by mixing 400 µg of nuclear extract proteins, 4 µg of the biotinylated DNA probe and 40 µl of streptavidin-conjugated agarose beads. The mixture was incubated at room temperature for 1 h with shaking, and centrifuged to pull down the DNA-protein complex. DNA-bound transactivators or p300 were dissociated and analyzed by Western blotting using antibodies specific for the candidate transactivators or p300.

*Western blot analysis*—Western blot analysis was performed as previously described [14]. In brief, proteins were separated by electrophoresis in a 4–15% sodium dodecyl sulfate-polyacrylamide gradient minigel (Bio-Rad, Hercules, CA) and electrophoretically transferred to a nitrocellulose membrane (Amersham Pharmacia Biotech, Piscataway, NJ). Western blots were probed with affinity purified rabbit polyclonal IgG at 1 µg/ml each against COX-2 (#4842), protein kinase C δ (PKCδ) (#2058) and phosphorylated PKCδ (#9374) from Cell Signaling Technology, Danvers, MA; and p300 (#sc-584), c-Jun (#sc-1694), CREB-2 (#sc-200), C/EBPβ (#sc-7962), p50 NF-κB (#sc-7178), p65 NF-κB (#sc-109), and control rabbit IgG (#sc-2027) from Santa Cruz Biotechnology, Santa Cruz, CA; and HIOMT (#AP4790c) from Abgent, San Diego, CA; or rabbit monoclonal IgG at 1 µg/ml against TPH-1 (#1915-1) from Epitomics, Burlingame, CA. Protein bands were detected by enhanced chemiluminescence (Amersham Pharmacia Biotech, Piscataway, NJ) and analyzed by densitometry using ImageJ software (National Institutes of Health, Bethesda, MD).

### Chromatin Immunoprecipitation (ChIP) assay

The assay was done as previously described [14] with minor modifications. In brief, 1% formaldehyde was added to cells and incubated for 20 min at 37°C. Cells were washed twice in phosphate-buffered saline, scraped, and lysed in lysis buffer (1% SDS, 10 mM Tris-HCl, pH 8.0 with 1 mM phenylmethylsulfonyl fluoride, pepstatin A, and aprotinin) for 10 min at 4°C. The lysate was sonicated, and the debris was removed by centrifugation. 1% volume of the lysate was used as DNA input reference. 10% volume of the lysate was diluted 10-fold with a dilution buffer (0.01% SDS, 1% Triton X-100, 1 mM EDTA, 10 mM Tris-HCl, pH 8.0, and 150 mM NaCl) followed by incubation with antibodies against the indicated transactivators, p300 or a non-immune rabbit IgG overnight at 4°C. Immunoprecipitated complexes were collected by using protein A/G plus agarose beads. The precipitates were extensively washed and incubated in an elution buffer (1% SDS and 0.1 M NaHCO_3_) at room temperature for 20 min. Cross-linking of protein-DNA complexes was reversed at 65°C for 5 h, followed by treatment with 100 µg/ml proteinase K for 3 h at 50°C. DNA was extracted three times with phenol/chloroform and precipitated with ethanol. The pellets were resuspended in TE buffer and subjected to quantitative PCR (qPCR) analysis, using an ABI 7900 real time qPCR system (Applied Biosystems, Foster City, CA). qPCR was performed according to the manufacturer's procedural manual. In brief, input DNA or ChIP-isoloated DNA was mixed with primers, SYBR Green mix and di-deionized water and placed in ABI 7900. C_t_ value of input DNA from SF-Fb or pFb with and without PMA treatment did not vary (Fig. 1A). Hence, we used the input DNA as a reference for normalization. The results were expressed as a ratio of ChIP DNA/input DNA. The primer sequences for the positive binding core promoter region are: 5′-forward GGT ATC CCA TCC AAG GCG A and 3′-reverse CAG GTT TCC GCC AGA TGT CT. The negative control primer sequences are: 5′-forward TAA CTT TTT TCC TTA TGC TC and 3′-reverse AGA GTG AAG AGT TCC ATG TA.

### Analysis of HAT activity

HAT activity was measured by an enzyme-immunoassay using a kit according to manufacturer's instruction (Upstate, Temecula, CA). p300 proteins were isolated from nuclear extracts by IP with anti-p300 antibodies (Santa Cruz Biotechnology, Santa Cruz, CA) and incubated with a reaction mixture containing 10 µl HAT assay buffer and 10 µl of acetyl-CoA (500 µM) at 30°C for 1 h. Tetramethylbenzidine substrate mixture was added and substrate acetylation was analyzed at 450 nm and 570 nm. The 570 nm value was subtracted from the 450 nm value. Histone H4 acetylation was included as positive controls.

### Transient p300 transfection

Fbs grown to 80-90% confluence were transfected with pCL vectors containing WT or ΔHAT p300 using lipofectamine 2000 as previously described [14]. In brief, 10 µg of plasmids were mixed with 25 µl of lipofectamine 2000 reagent, and the mixture was slowly added to cells cultured in a 10-cm dish. After incubation for 8 h, cells were incubated in fresh medium containing 10% FBS for 18 h.

### SiRNA transfection

Human TPH-1 and HIOMT siRNA composed of three different sequences were purchased from Santa Cruz Biotechnology. The siRNA sequences used to silence human THP-1 are: 5′-CUG UGA AUC UAC CAG AUA ATT-3′; 5′-CCA ACA GAG UUC UGA UGU ATT-3′ and 5′-GGA AUG UCU UAU CAC AAC UTT-3′. The siRNA sequences used to silence human HIOMT are: 5′-CUG UCA GUG UUC CCA CUU ATT-3′; 5′-CUG UAC CCU GGA UGU AAG ATT-3′ and 5′-GAG AGG AUC UAC CAC ACU UTT-3′. Human PKCδ siRNA (5′-CCA UGA GUU UAU GGC CAC CTT-3′) were purchased from Santa Cruz Biotechnology, Santa Cruz, CA. A control scrambled RNA (scRNA) with a sequence of 5′-UUC UCC GAA CGU GUC ACG UTT-3′ was included as control. Transfection of siRNA was performed as previously described [14]. In brief, siRNA or scRNA were mixed with lipofectamine 2000 at room temperature for 20 min and the mixture was added to SF-Fb or pFb dropwise and incubated for 8 h.

### Preparation of CMF2 fraction

CMF2 fraction was prepared from fibroblast CM or control medium as previously described [6]. In brief, cultured medium was filtered through a 10 kDa ultra filtration membrane (Millipore, Bedford, MA). The <10 kDa fraction (<10K) with positive COX-2 inhibitory activity was lyophilized and suspended in di-deionized water (ddH_2_O). The reconstituted <10K fraction extracted with methanol and the extract was chromatographed on a Hiload 16/60 Superdex 30 column (16×600 mm) (Amersham, Piscataway, NJ) in a FPLC system and eluted with phosphate saline buffer, pH 7.4. Fractions of each peak were collected and reconstituted to original concentrations. Their effect on COX-2 protein levels was evaluated using extensively washed SF-Fb as test cells. Fractions collected from the second peak (CMF2) of pFb were found to be active in inhibiting COX-2 expression.

### Metabolomic analysis

Metabolites in the CMF2 fraction was analyzed using ultrahigh performance liquid chromatography (Acquity UPLC system, Waters Corporation, Milford, MA) coupled with a quadrupole time of flight mass spectrometer (QTof MS) (Xevo, Waters) as previously described [6]. In brief, UPLC separation was carried out at 40°C with a flow rate of 0.2 ml/min using water/formic acid and acetonitrile/formic acid gradient [6]. QTof MS was operated in a positive electrospray ionization mode. As we suspected that cytoguardins are small molecules containing indole moieties [5], we acquired data by MassLynx software (Waters) in uncentroided format over a mass range of m/z 100–800 with scan duration of 0.5 sec and an interscan delay of 0.1 sec. For accurate mass measurement, the QTof MS was calibrated with formic acid 0.1% (vol/vol) in acetonitrile/water (50∶50, vol/vol) and the dynamic range enhancement mode was used for data recording. Raw data of all samples were processed using MassLynx software.

### 5-MTP enzyme immunoassay (EIA)

The assay was modified from a previously described method [6]. Microtiter plates (96-well) were coated with 5-MTP standards or samples in coating buffer (0.05 M carbonate-bicarbonate, pH 9.6) at 4°C overnight. The coating buffer was removed and the plate was washed five times with 200 µl TBST. After the plate had been blocked with 100 µl of 1% BSA/TBS and washed, 100 µl of a rabbit polyclonal anti-5-MTP antibody (Abcam, Cambridge, UK.) was added to each well and incubated for 1.5 h at room temperature or overnight at 4°C. The antibody is highly specific for 5-MTP with little cross-reactivity with related compounds such as 5-hydroxytryptophan, L-tryptophan and 5-methoxytryptamine [6]. The plate was washed and treated with biotinylated anti-rabbit IgG (Abcam, Cambridge, UK) followed by streptavidin-HRP (Upstate, Temecula, CA). Tetramethylbenzidine (TMB) (Upstate) substrate mixture was added and the optimal density (O.D.) was analyzed at 450 nm and 570 nm. For each experiment, a standard curve was generated using pure 5-MTP.

### Statistical Analysis

Values were expressed as mean ± SEM. Differences between groups were analyzed using One Way ANOVA with SigmaStat software (Systat Software, Inc.). *P*<0.05 was considered statistically significant.
